# Supplementary material for: Mechanisms governing the pioneering and redistribution capabilities of the non-classical pioneer PU.1
Source: Nat Commun. 2020 Jan 21;11:402. doi: 10.1038/s41467-019-13960-2 (PMC6972792; doi:10.1038/s41467-019-13960-2)
Supplement: Supplementary file 7 — Source data [file 41467_2019_13960_MOESM7_ESM.zip › Source_Data/FigureS2/FigureS2B_MotifScanOutput/KmeansCluster11/homerResults/motif1.similar.html]

motif1

## Information for motif1

T
C
A
G
C
G
T
A
T
A
C
G
T
C
G
A
C
T
A
G
T
C
A
G
G
C
T
A
C
G
T
A
T
A
C
G
G
A
C
T
T
A
C
G
  
Reverse Opposite:  

A
T
G
C
C
T
G
A
A
G
T
C
G
C
A
T
C
G
A
T
A
G
T
C
A
G
T
C
A
G
C
T
A
T
G
C
G
C
A
T
A
G
T
C
  

|  |  |
| --- | --- |
| p-value: | 1e-1277 |
| log p-value: | -2.941e+03 |
| Information Content per bp: | 1.623 |
| Number of Target Sequences with motif | 2688.0 |
| Percentage of Target Sequences with motif | 82.73% |
| Number of Background Sequences with motif | 8127.8 |
| Percentage of Background Sequences with motif | 17.98% |
| Average Position of motif in Targets | 102.6 +/- 34.4bp |
| Average Position of motif in Background | 100.4 +/- 68.2bp |
| Strand Bias (log2 ratio + to - strand density) | 0.1 |
| Multiplicity (# of sites on avg that occur together) | 1.33 |
| Motif File: | file (matrix) reverse opposite |

### Similar de novo motifs found

|  |  |  |  |  |  |  |  |
| --- | --- | --- | --- | --- | --- | --- | --- |
| Rank | Match Score | Redundant Motif | P-value | log P-value | % of Targets | % of Background | Motif file |
| 1 | 0.979 | T C G A C T G A T A C G T C G A A C T G A C T G C G T A C G T A T C A G A G C T T A C G T C A G | 1e-1175 | -2707.652321 | 73.81% | 13.78% | motif file (matrix) |
| 2 | 0.966 | C T G A T C A G C T G A T A C G T C G A A C T G A C T G C G T A C G T A T C A G G A C T T A C G T C A G | 1e-1159 | -2670.362228 | 73.93% | 14.13% | motif file (matrix) |
| 3 | 0.974 | C T G A T C A G T C G A T C A G C T A G C G T A G C T A T C A G G A C T T A C G | 1e-1147 | -2641.700860 | 73.93% | 14.34% | motif file (matrix) |
| 4 | 0.955 | T G C A A G T C T A G C C G T A A G T C C G A T A C G T A G T C A G T C A G C T A T G C G A C T A G C T G A C T | 1e-1109 | -2554.959840 | 69.56% | 12.39% | motif file (matrix) |
| 5 | 0.980 | A T G C C T G A A T G C C G A T C G A T G T A C A G T C A G C T A T G C | 1e-1080 | -2488.474657 | 79.22% | 19.26% | motif file (matrix) |
| 6 | 0.883 | T C G A A G T C G A C T G C A T A G T C A T G C A C T G A T G C G A T C G A T C | 1e-921 | -2122.983957 | 79.38% | 23.05% | motif file (matrix) |
| 7 | 0.940 | A T G C C G T A A G T C A C G T A C G T A G T C A G T C A G C T | 1e-870 | -2004.334493 | 58.11% | 10.17% | motif file (matrix) |
| 8 | 0.912 | A T G C C T G A A G T C G C A T G A C T A G T C A G T C A C G T | 1e-802 | -1847.694777 | 83.81% | 30.49% | motif file (matrix) |
| 9 | 0.923 | C G T A A G T C A C G T A C G T A G T C A G T C A C G T | 1e-798 | -1839.110612 | 47.92% | 6.57% | motif file (matrix) |
| 10 | 0.851 | A T G C C G A T C A G T G A T C G A T C A C T G A T G C G A C T A G C T G C A T | 1e-650 | -1498.147465 | 70.79% | 23.46% | motif file (matrix) |
| 11 | 0.898 | C T G A A G T C G C A T A C G T G A T C A G T C A C G T C A G T A G C T | 1e-643 | -1482.586864 | 70.05% | 23.07% | motif file (matrix) |
| 12 | 0.921 | C T G A A T G C C G A T A C G T A G T C A G T C A C G T | 1e-643 | -1481.268916 | 89.69% | 42.92% | motif file (matrix) |
| 13 | 0.903 | A G C T A T G C C A G T A T C G T A C G A C T G A C T G C G T A C G T A A C T G C G A T T C A G A T G C | 1e-597 | -1375.958163 | 72.55% | 26.61% | motif file (matrix) |
| 14 | 0.865 | A T C G C G A T A C G T A G T C A T G C A C T G A T G C G C A T | 1e-577 | -1330.884826 | 74.12% | 28.67% | motif file (matrix) |
| 15 | 0.775 | A G T C C G T A A G T C A C G T A C G T A G T C A G C T | 1e-563 | -1296.597356 | 47.15% | 10.12% | motif file (matrix) |
| 16 | 0.926 | C T G A C G T A T A C G T G C A C T A G T C A G G T C A C T G A A T C G C A G T A G C T T G C A | 1e-505 | -1164.811664 | 48.72% | 12.27% | motif file (matrix) |
| 17 | 0.822 | C G A T A C G T G T A C T A G C A C T G A T G C G A C T A G C T G A C T | 1e-504 | -1162.535124 | 65.68% | 24.04% | motif file (matrix) |
| 18 | 0.753 | A T C G A C G T A C G T A G T C A G T C A T G C A G T C | 1e-428 | -987.189737 | 63.07% | 24.67% | motif file (matrix) |
| 19 | 0.836 | T C G A C G A T A T C G T C G A C T A G T C A G T G C A C T G A T A G C C G A T A T C G T A G C T G A C | 1e-384 | -886.141431 | 42.84% | 11.93% | motif file (matrix) |
| 20 | 0.822 | A C G T C T G A C A T G A T C G A C G T A G C T A T G C G T A C A C G T A T G C G A T C A G C T | 1e-369 | -851.892795 | 51.92% | 18.35% | motif file (matrix) |
| 21 | 0.842 | G C T A T G A C G A C T G T C A A T C G A C T G C G T A C G T A A C T G A C G T T C A G T C A G A G T C | 1e-295 | -680.740965 | 16.84% | 1.70% | motif file (matrix) |
| 22 | 0.761 | T C G A T C A G T C G A T C G A C T G A T C A G T C A G T C A G T C A G T G C A C G T A T A G C G A T C A T G C | 1e-265 | -611.293356 | 37.86% | 12.55% | motif file (matrix) |
| 23 | 0.856 | A G C T T A C G A C T G A T C G C T G A G T C A C T A G A C G T C T A G G T C A T C A G T A G C | 1e-232 | -535.255444 | 19.11% | 3.40% | motif file (matrix) |
| 24 | 0.824 | A G T C A C G T A C G T A G T C A G T C A C G T A C T G | 1e-204 | -470.613953 | 30.07% | 9.83% | motif file (matrix) |
| 25 | 0.772 | C T A G C T G A C T G A T C G A C T G A T A G C C T A G C T A G T C G A C G T A T A C G C A G T A T C G | 1e-202 | -465.769590 | 19.58% | 4.20% | motif file (matrix) |
| 26 | 0.792 | A G T C T G C A G T C A A G T C C G T A C T A G T C A G T G C A C G T A T A C G A C G T A C G T C T G A | 1e-161 | -371.717359 | 10.37% | 1.24% | motif file (matrix) |
| 27 | 0.844 | G A T C A G T C G A C T A T C G A G T C A C G T A C G T A G T C A G T C C A G T T A G C G C A T A C G T A G C T | 1e-144 | -333.653840 | 27.39% | 10.46% | motif file (matrix) |
| 28 | 0.816 | C T G A T C G A C T G A T A C G C T G A A C T G T C A G C G T A T C G A T C A G A T G C T G A C T G C A T G A C | 1e-119 | -275.840716 | 14.56% | 3.82% | motif file (matrix) |
| 29 | 0.809 | C T G A A C G T A G C T A G C T C A G T C A T G T C A G C T A G T C A G T C G A C T G A T A C G C G A T T A C G | 1e-93 | -214.351699 | 14.47% | 4.62% | motif file (matrix) |
| 30 | 0.837 | A T G C G A C T A T G C A C G T A C G T A G T C A G T C A G C T A T G C G A C T A G C T A G C T | 1e-81 | -186.579526 | 17.42% | 6.93% | motif file (matrix) |
| 31 | 0.823 | C A T G C T A G C A T G A C T G C T A G A T C G C T A G T C A G T G C A C T G A T C A G A G C T A T C G | 1e-74 | -170.783248 | 6.62% | 1.23% | motif file (matrix) |
| 32 | 0.770 | G T C A A C T G A C T G C G T A C G T A A G T C A C G T A C T G A T G C | 1e-34 | -80.089527 | 6.16% | 2.11% | motif file (matrix) |
| 33 | 0.765 | A C G T C G T A A C T G A C T G G T C A C G T A A C T G C G A T A T C G C A T G A C G T T C A G | 1e-19 | -44.813000 | 1.17% | 0.13% | motif file (matrix) |
